# Post-renal Transplantation Triple Neuralgia: A Case Report

**DOI:** 10.7759/cureus.49518

**Published:** 2023-11-27

**Authors:** Arun Kalava, Assad A Khan, Simeon V Mihaylov

**Affiliations:** 1 Anesthesiology, University of Central Florida College of Medicine, Orlando, USA; 2 Medicine, University of Central Florida College of Medicine, Orlando, USA; 3 Pain Management, TampaPainMD, Tampa, USA

**Keywords:** renal transplant, peripheral nerve stimulation, neuralgia, gate theory, chronic pain

## Abstract

Renal transplantation is the most common solid organ transplant in the United States. Post-transplant neuralgia is a frequent complication and may be due to infection, medication side effects, post-transplant lymphoproliferative disorder (PTLD), or the procedure itself. This case report describes an instance of post-renal transplant neuralgia in a 70-year-old Caucasian female. Diagnostic nerve blocks revealed the involvement of the ilioinguinal, genitofemoral, and lateral femoral cutaneous nerves. The case report details management that included nerve blocks, radiofrequency ablation (RFA), and a permanent peripheral nerve stimulator (PNS) implant.

## Introduction

Renal transplantation is the most common solid organ transplant in the United States. Annually, over 17,000 kidney transplants are performed and over 100,000 people remain on the transplant waitlist. This number has grown in recent years, as it has become increasingly clear that transplantation improves measures of mortality and quality of life for those with end-stage renal disease (ESRD) [1]. Despite this benefit, the transplant process itself can result in the development of neurological complications from a variety of etiologies [1]. For all etiologies, the incidence of neuropathy seems to be higher among women, diabetics, hypertensive patients, and those with graft rejection episodes [2].

Following transplantation, there is an increased risk of infection due to the immunosuppression required to prevent transplant rejection. Central nervous system (CNS) infection is a significant source of neurological complications following solid organ transplant. Symptoms can be non-specific (i.e. headache, fever, or altered mental status) or more suggestive of neurological involvement (i.e. focal neurological deficits, coma). In addition to antibiotic treatment of the infection, cessation of the immunosuppressive regimen should also be considered [1]. Additionally, some of the medications that are used to induce an immunosuppressed state have the potential to cause peripheral neuropathy. In most of these cases, the symptoms of peripheral neuropathy dissipate upon cessation of the medication [1].

Solid organ transplant patients are at a four-fold increased risk of malignancy compared to the general population. This is most likely due to increased susceptibility to infection by oncogenic pathogens due to the immunosuppressed state of these patients. Following skin cancer, the most common malignancy in post-transplant patients is post-transplant lymphoproliferative disorder (PTLD) [1]. The particular manifestations of PTLD depend on the site of tumor formation. This typically occurs within lymph nodes and can cause localized symptoms due to compressive effects. Compression of nerves can result in neuralgia with associated burning, pain, numbness, and/or paresthesias [3].

Neuropathy can also develop as a result of techniques used during the surgery itself. Damage to nerves adjacent to the target tissue or organ can occur during the operation. This can be due to the transection of nerve fibers, as a result of prolonged retraction resulting in compression of the nerve or due to ischemia of the nerve due to interruption of blood supply during the procedure (i.e. clamping of vessels) [1]. The risk of transection can be reduced via an increased emphasis on the understanding of anatomy within the operative field and conscious identification of nearby nerves during the operation [4]. Limiting the duration of surgery to the extent that it does not compromise surgical technique and accuracy reduces the length of time of retraction and may reduce the incidence of neuropathy from prolonged compression.

This case report aims to add to the growing body of evidence on the incidence of post-transplant neuralgia. We present a patient who had an immediate onset of right-sided suprapubic, groin, and anterolateral thigh pain following a renal transplant. We also discuss the use of nerve blocks, radiofrequency ablation (RFA), and peripheral nerve stimulators (PNS) as potential therapeutic interventions for the treatment of refractory post-transplant neuralgia.

## Case presentation

**Figure 1:**
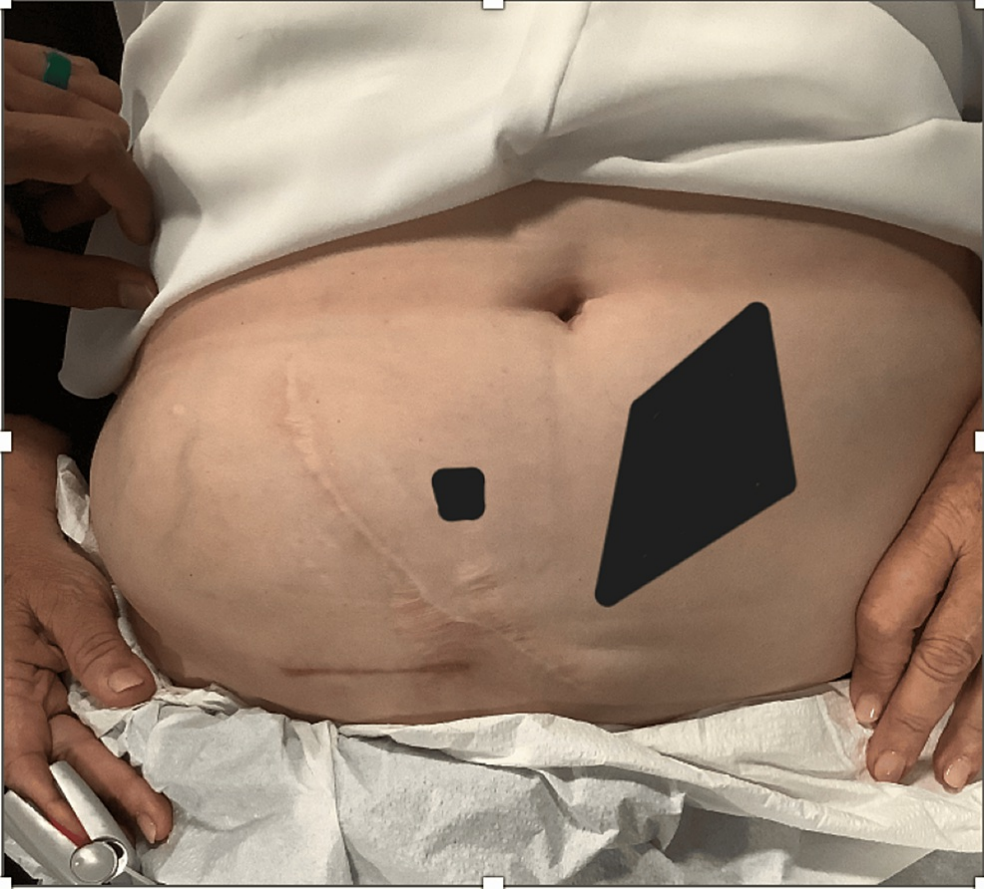
Surgical scar from the renal transplant

A 70-year-old Caucasian female presented to our clinic with right abdominal pain. The pain started four years prior to seeking care in our clinic, immediately after a renal transplant. The reason for renal transplant in this patient was chronic kidney disease, which progressed to the point of renal failure. The pain started at the incision scar and radiated to the suprapubic and right groin area and was constant, sharp, and burning in nature. The patient also reported anterolateral thigh pain, stopping at the level of the knee. On initial inspection, the patient appeared obese and had a well-healed right abdominal scar. (Figure 1).

Before presenting to our clinic, the patient had tried physical therapy, acupuncture, and steroid injections at the site. None of these treatments provided pain relief. Three years after the onset of pain, the patient underwent a right ilioinguinal nerve resection. This was also insufficient in managing the patient's symptoms. She reported taking 5 mg of oxycodone daily at night to help her sleep.

A diagnostic ultrasound-guided right ilioinguinal nerve block was performed with 15 mL bupivacaine 0.5% and 10 mg dexamethasone. The procedure provided more than 50% abdominal pain relief in the office; however, there was residual pubic and thigh pain. To address this, an ultrasound-guided right genito-femoral nerve block was done at the two-week follow-up appointment. The nerve was injected with 7 mL bupivacaine 0.5% and 10 mg dexamethasone. This provided more than 50% pain relief in the suprapubic and groin areas but did not alleviate the anterolateral thigh pain.

Follow-up done in two weeks revealed sustained pain relief from the ilioinguinal and genitofemoral nerve blocks. A right lateral femoral cutaneous nerve (LFCN) block was performed to address her antero-lateral thigh pain. One month later, the right LFCN block was repeated, as the patient could not ascertain whether it had helped the first time. The procedure provided immediate pain relief, which continued for one week. On the next visit two weeks later, a right LFCN radio-frequency ablation was performed at 80 degrees Celsius over 2 minutes. Two days following the procedure, the patient experienced soreness and a burning sensation in the area, suggestive of post-radio-frequency neuritis. To address this, a right LFCN block was done, which provided immediate pain relief lasting for 24 hours.

**Figure 2:**
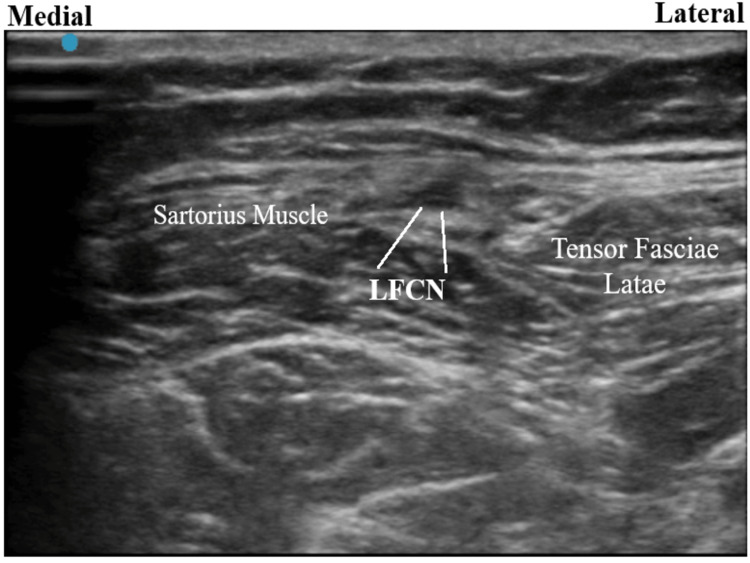
Ultrasound image of the lateral femoral cutaneous nerve

Follow-up was done 14 days after the right LFCN block, and the patient agreed to a PNS trial targeting the right LFCN. This provided more than 80% immediate pain relief. Due to the efficacy of the temporary treatment, the patient agreed to a permanent PNS implantation. For the procedure, the patient was placed in a supine position and the area was prepared in a sterile manner; 1% lidocaine was used to anesthetize the skin. A 15-blade scalpel was used to make a 2 mm stab incision. A Stimrouter (Bioventus LLC, Durham, NC, USA) PNS implantation was done after confirmation of sensory stimulus in the area of the targeted nerve under direct ultrasound visualization (Figure 2). 3-0 vicryl sutures were then used to close the incision. Dermabond (Ethicon Inc., Cincinnati, OH, USA) was applied, along with Steri-Strips (3M LLC, Saint Paul, MN, USA) to help seal the wound. An external pulse generator was then programmed to the level of stimulation that provided the most pain relief. At the two-month follow-up over the phone, the patient reported having used the device consistently since its implantation. For the majority of this time, she did not experience any pain relief. However, she reported significantly decreased pain in the days leading up to this phone call and did not feel the need to use the stimulator during this period.

## Discussion

Quality of life is strongly influenced by the presence of chronic pain and the incidence of the latter after renal transplantation is estimated to be approximately 62% [5]. Zorgdrager M et al. performed a cohort analysis involving 199 patients which showed that 32% of patients reported inguinal pain post-renal transplant. Of those, only 24% sought targeted pain management [4]. Even though the incidence of chronic inguinal pain is common after renal transplantation, it is underrepresented in scientific studies.

Targeting a multiplex pain syndrome requires a comprehensive stepwise approach. Having undergone an abdominal surgery such as renal transplantation places the patient at a much higher risk of ilioinguinal entrapment syndrome than the general population [6]. We performed a diagnostic right ilioinguinal nerve block. Pain relief greater than 50% following the nerve block, as seen in this patient, is sufficient for confirming the involvement of the blocked nerve [6].

The presence of residual inguinal and pubic pain prompted us to perform a diagnostic genitofemoral nerve block, another commonly affected nerve following abdominal surgery in the inguinal area [7]. This resolved our patient’s suprapubic and groin pain but did not alleviate the lateral thigh pain. 

Along with the ilioinguinal and genitofemoral, another commonly affected nerve after inguinal surgery is the LFCN [8]. Damage to this nerve could easily explain our patient’s lateral thigh pain. Consequently, we performed two consecutive LFCN blocks. One week of pain relief following the second nerve block confirmed the involvement of the LFCN in the patient’s condition [6]. 

The ilioinguinal and genitofemoral nerve blocks provided sustained pain relief in the groin and suprapubic area. As such, we decided to cease the interventions that targeted the aforementioned nerves until symptoms developed. However, the LFCN block provided only temporary relief which prompted us to perform a lateral femoral cutaneous nerve RFA with the intention of achieving long-term pain relief. Two days following the procedure the patient developed symptoms of post-RFA neuritis. A study done by Halena M Gazelka et al. on the frequency of neuropathic pain after radiofrequency denervation of the third occipital nerve concluded that 19% of patients experience such a complication; 83% of these patients require active treatment [9]. Because less invasive measures proved ineffective, we proceeded to PNS of the left LFCN.

Peripheral nerve stimulation is based on the gate control theory proposed by Melzack and Wall in 1965 [10]. According to gate theory, large diameter non-nociceptive sensory Aβ nerve fibers carry signals to interneurons located in the dorsal horn of the spinal cord. These signals are thought to inhibit transmission of nociceptive signals from smaller diameter C fibers, effectively closing the pain “gate”. This mechanism effectively blocks afferent pain signals from traveling from the spinal cord to the higher CNS centers. PNS may also alter the release of endogenous neurotransmitters involved in analgesia and modulation of central circuits. These neurotransmitters include but may not be limited to norepinephrine, Arc protein, and GluA1. Additionally, PNS stimulation affects the plasticity of NMDA pathways which are known to be involved in pain associated with peripheral nerve injury [11]. As a result of these interactions, there is a possibility for diminished or completely absent sensation of pain. A case-control study involving 94 patients concluded that over 60% of the patients who underwent PNS device placement experienced long-term pain relief [12].

## Conclusions

Neuropathic pain following renal transplantation is a much more common issue than previously thought and should not be overlooked by the managing physicians. Commonly affected nerves are the ilioinguinal/iliohypogastric, genitofemoral, and lateral femoral cutaneous nerves. A multidisciplinary team including a pain physician should be formed to tend to patients’ pain symptoms following transplantation. Feasible options for managing pain include a nerve block, RFA, or PNS if conservative therapy fails or is inadequate.
